# Single-cell measurements of two-dimensional binding affinity across cell contacts

**DOI:** 10.1016/j.bpj.2021.10.010

**Published:** 2021-10-13

**Authors:** Manto Chouliara, Victoria Junghans, Tommy Dam, Ana Mafalda Santos, Simon J. Davis, Peter Jönsson

**Affiliations:** 1 Department of Chemistry, Lund University, Lund, Sweden; 2 Radcliffe Department of Medicine and Medical Research Council Human Immunology Unit, John Radcliffe Hospital, University of Oxford, Oxford, United Kingdom

## Abstract

The two-dimensional (2D) affinity between protein molecules across contacting cells is a key parameter regulating and initiating several cellular processes. However, measuring 2D affinity can be challenging, and experimental data are limited. In addition, the obtained 2D affinities are typically averaged over the cell population. We here present a method to measure 2D affinity on single cells binding to polyhistidine-tagged fluorescent ligands anchored to a supported lipid bilayer (SLB). By decreasing the density of ligands in the SLB using imidazole, a new steady-state accumulation in the contact is obtained, and from this change, both the 2D affinity and the number of receptors on the cell can be determined. The method was validated on an SLB containing rat CD2 binding to the rat CD48 mutant T92A expressed on Jurkat T cells. The addition of imidazole did not influence the average 2D affinity (1/*K*_d_), and the spread in affinities within the cell population was low, *K*_d_ = 4.9 ± 0.9 molecules/*μ*m^2^ (mean ± SD), despite an order of magnitude spread in ligand accumulation because of differences in receptor density. It was also found that cell contact size increased both with ligand density and with the number of receptors per cell but that the contact size stayed approximately constant when lowering the ligand density, above a density of around 10 rat CD2 molecules/*μ*m^2^, after the contact first had formed, indicative of a heterogeneous process. In summary, this method not only allows for single-cell affinities to be measured, but it can also reduce measurement and analysis time and improve measurement accuracy. Because of the low spread in 2D *K*_d_ within the cell population, the analysis can further be restricted to the cells showing the strongest binding, paving the way for using this method to study weak binding events.

## Significance

The binding of molecules across contacting cells is important for several biological processes. However, the magnitude of these interactions is often estimated from solution measurements that lack the natural two-dimensional constraints of the cell-cell contacts in vivo. We here show how two-dimensional affinities can be measured on individual cells. One of the contacting cells is replaced by a supported lipid bilayer containing polyhistidine-anchored ligands that in turn bind to receptors on cells. By titrating the ligand concentration in the contact with imidazole, both the affinity and the receptor density on the cell can be determined from the accumulation of fluorescently labeled ligands. In addition to providing single-cell information, the presented method also showed high sensitivity and the ability to reduce measurement and analysis time considerably.

## Introduction

The interaction between T cell receptors (TCRs) on a T cell and their cognate antigen bound to major histocompatibility complexes (MHCs) on the surface of an antigen-presenting cell is the first step of an adaptive immune response. Because of the significance of this interaction, there has been extensive research focusing on the molecular mechanisms leading to T cell activation and downstream signaling (1, 2, 3). It has been argued that the binding kinetics of the TCR-antigen MHC interaction constitutes the basis for the necessary selectivity, specificity, and sensitivity that characterize the adaptive immune response (4, 5, 6). In addition, several other interactions between receptors and ligands across cell-cell contacts have been found important for regulating the activation of the adaptive immune response (7,8). Traditionally, the binding kinetics of these interactions have been estimated from measurements between receptors anchored to a biosensor surface with ligands binding from the bulk solution (9,10). However, these three-dimensional affinities do not reflect the natural lateral confinement of the molecules on a cell membrane nor consider the effects of ligand diffusion or cellular dynamics of binding across cell-cell contacts in vivo (4,11, 12, 13). Therefore, three-dimensional affinities cannot in general be directly converted into physiologically relevant two-dimensional (2D) affinities (4,14,15), although recent studies on model systems have made progress in understanding how cooperative interactions (16) and membrane fluctuations (17) influence this.

Measurements of 2D affinities and lifetimes have typically been made using either mechanical-based (18, 19, 20) or fluorescence-based methods (21, 22, 23). Different micropipette adhesion assays that periodically bring two cells, or a cell and a bead, in contact belong to the former and have been used to both estimate the 2D lifetime as well as the 2D affinity of different ligand-receptor interactions (4,24,25). However, only relative affinities can be obtained with this approach because of difficulties in measuring the contact area between the two adhering cells. In addition, this method cannot be used together with auxiliary adhesion molecules to stabilize the bonds or to measure at higher ligand densities. Fluorescence-based methods do not suffer from these constraints and have been employed to study the 2D binding kinetics, especially in the contact between live immune cells and model cell membranes called supported lipid bilayers (SLBs) (22,26,27). This includes single-molecule fluorescence microscopy, which mainly has been used to measure 2D lifetimes but also to obtain 2D affinities (28,29). However, a potential drawback with this approach is that not all cell receptors are able to bind to the fluorescently labeled ligands in the SLB because of cell surface undulations, and it is therefore possible that the number of available receptors in the contact is overestimated. To measure the 2D affinity of, in particular, weak interactions with a higher concentration of ligands in the SLB, the fluorescence-based Zhu-Golan method has been the dominant technique (18,19). According to this method, SLBs functionalized with different ligand densities are made, and the 2D affinity can be obtained from the change in accumulation of ligands in the cell-SLB contacts at the different ligand densities (26). However, there is generally a significant spread in accumulation over the cell population, and averaging over numerous contacts for each ligand concentration is therefore required to obtain accurate average values for the affinity. This can be a time-consuming procedure in which any natural distribution of affinities within the cell population will also be lost.

To address these issues, we here extend the classical Zhu-Golan technique and present a method for measuring 2D affinities of ligand-receptor interactions on individual cells. As an additional benefit, this method also gives the number of mobile receptors per cell, which was used to relate how the cell-SLB contact size and the maximal bound ligand density in the contact depended on this number. As a model system, we used Jurkat T cells transduced with the rat CD48 mutant T92A (rCD48_T92A_) that binds to fluorescently labeled rat CD2 (rCD2) coupled to an SLB using a polyhistidine tag at the protein’s C-terminus. CD2 is an adhesion molecule that is critical for aligning the T cell and antigen-presenting cell surfaces helping the TCR-MHC interaction (30,31). The rCD2-rCD48_T92A_ interaction was selected as it has previously been characterized using the Zhu-Golan method yielding a 2D dissociation constant (affinity^-1^), *K*_d_, of 6 molecules/*μ*m^2^ (27,32), similar to the 2D affinity of the corresponding adhesion pairs in humans, i.e., human CD2 binding human CD58 (21), and also that of TCR binding cognate antigen-presenting MHCs (27,33). To obtain single-cell affinities, rCD48_T92A_-transduced Jurkat T cells were allowed to bind to an SLB with a high rCD2 concentration, resulting in visible accumulation of fluorescently labeled rCD2 in the cell-SLB contacts. The subsequent addition of imidazole reduced the amount of the polyhistidine-tagged rCD2 on the SLB, resulting in a shift of the bound/free ligand ratio in the cell-SLB contacts. From this shift, both the binding affinity and the number of mobile cell receptors could be determined using the classical Zhu-Golan analysis method (21) but for individual cells instead of an average over the entire cell population. Although the receptor density varied by approximately an order of magnitude in the cell population, there was no significant difference in the affinities between cells expressing low or high amounts of rCD48_T92A_, and a 2D *K*_d_ of 4.9 ± 0.9 molecules/*μ*m^2^ (mean ± SD) was found for the rCD2-rCD48_T92A_ interaction among the subgroups. It is thus possible to measure 2D *K*_d_ values with high precision on individual cells, opening up for affinity studies in which the binding is expected to be heterogenous among the cell population, at the same time as providing a highly accurate method to study weak binding events.

## Materials and methods

### Vesicles and SLB formation

Vesicle solutions containing 90 weight percentage 1-palmitoyl-2-oleoyl-*sn*-glycero-3-phosphocholine (Avanti Polar Lipids, Alabaster, AL) and 10 weight percentage 1,2-dioleoyl-*sn*-glycero-3-[(*N*-(5-amino-1-carboxypentyl)iminodiacetic acid)succinyl] (nickel salt) (Avanti Polar Lipids) were prepared at a total lipid concentration of 0.5 mg/mL as previously described (27). Initially, the lipids were mixed with 100 *μ*L of chloroform, then the chloroform was evaporated using a N_2_ gas flow for ∼5 min, and the dried lipids were resuspended and mixed in 1 mL of filtered HEPES-buffered saline (HBS) buffer (150 mM NaCl and 10 mM 2-[4-(2-hydroxyethyl)piperazin-1-yl]ethanesulfonic acid (Sigma-Aldrich, St. Louis, MO) (pH 7.4)). The vesicles were incubated on ice for 1 h followed by tip sonication with a CV18 model tip sonicator (Chemical Instruments, Stockholm, Sweden) for 15 min with a pulse time of 10 s and an amplitude of 40%. The small unilamellar vesicles were stored at 4°C and were made fresh every month.

SLBs were made using the method of vesicle fusion and rupture as previously described (27,34). In more detail, 0.15 mm-thick, round glass slides (number one coverslips, Ø 25 mm; Thermo Fisher Scientific, Waltham, MA) were cleaned for 30 min in 80°C heated piranha solution (75% sulfuric acid (99.9%, Sigma-Aldrich) and 25% hydrogen peroxide (30%, Sigma-Aldrich)). The glass slides were then rinsed meticulously with deionized water and dried with N_2_ gas. Afterwards, a single silicon well (Silicon isolators, 12 × 4.5 mm diameter, 1.7 mm depth; Grace Bio-Labs, Bend, OR) was cleaned with 99% ethanol and Milli-Q water and paper dried. Furthermore, any potential dust particles were removed using adherent tape. The silicon well was attached to a piranha-cleaned glass slide and placed in an Attofluor Cell Chamber (Thermo Fisher Scientific). Vesicles diluted 1:10 in 30 *μ*L HBS buffer were added to the well and were incubated for 1 h at room temperature (RT). The incubation time allowed the vesicles to adsorb to the glass surface, rupture, and create a continuous and fluid SLB. After 1 h, the nonruptured vesicles were washed away with filtered HBS buffer.

### Cell lines and culture

Human E6.1 Jurkat T cells were transduced using a lentivirus to express rCD48_T92A_, a strong binding mutant of rCD48 with a 2D *K*_d_ of 6 ± 1 molecules/*μ*m^2^ (mean ± SD) as described previously by Junghans et al. (27). The 2D affinity for rCD2 binding rCD48_T92A_ is similar to that measured for human CD2 binding human CD58 (21,35) and ∼6 times lower than that for rCD2 binding wild-type rCD48 (26). The Jurkat T cells were maintained in Roswell Park Memorial Institute (RPMI) 1640 medium (Sigma-Aldrich), which was supplemented with 10% fetal bovine serum (Sigma-Aldrich), 2% L-glutamine (Sigma-Aldrich), 1% sodium pyruvate (Sigma-Aldrich), 1% HEPES (Sigma-Aldrich), and 1% penicillin-streptomycin (Sigma-Aldrich). The cells were kept in an incubator at 37°C in a humidified atmosphere with 5% CO_2_ and were subcultured every 2–3 days.

### Affinity measurements

Recombinant rCD2 was produced as previously described (26,36) and was genetically modified at the C-terminus with a double histidine tag (12 × H), allowing the proteins to bind to 1,2-dioleoyl-*sn*-glycero-3-[(*N*-(5-amino-1-carboxypentyl)iminodiacetic acid)succinyl] (nickel salt) lipids in the SLB. For the binding affinity measurements, 3 *μ*g/mL of rCD2 labeled with Alexa Fluor 647, using an Alexa Fluor antibody labeling kit from Thermo Fisher Scientific, was added to the well and was left to incubate at RT. After 40–50 min of incubation, unbound proteins were washed away with filtered HBS buffer before adding 20–25 *μ*L of cell media solution containing rCD48_T92A_-expressing Jurkat T cells. Contact growth reached an equilibrium after 40 min of incubation. Subsequently, fluorescent and brightfield images of numerous cell-SLB contacts were captured while saving their exact location on the SLB. After the imaging process, 3–8 *μ*L of a 100 mM imidazole solution was gradually added to the well, resulting in an imidazole concentration of 5–13 mM inside the well. After an incubation period of ∼30–60 s, when the free rCD2 density had approximately halved compared with its initial concentration, the SLB was washed eight times with 70 *μ*L of filtered HBS buffer to rinse away any traces of imidazole. When incubating the SLB with imidazole for longer times, the cell contact area starts to reduce in size as the ligand density decreases (*F* ∼10 molecules/*μ*m^2^), and the cells could subsequently be washed off (Fig. S1; Video S1). Previous cell studies have shown that even 12-h incubation with similar concentrations of imidazole only moderately influences cell viability (37). The short incubation period used here is therefore not expected to significantly influence cell viability. Removing the imidazole concluded the decrease of the ligand concentration, and the cell-SLB contacts were incubated once again for 40 min after which the same cell-SLB contacts were imaged anew. The above-mentioned procedure was repeated three times, yielding a total of four different ligand concentrations for each contact. Successful SLB formation is marked by lateral mobility in the functionalized proteins. This was verified using fluorescence recovery after photobleaching (FRAP) before each experiment (Video S2). The diffusion coefficient, *D*, and the immobile fraction, *γ*_0_, were measured to 1.5 ± 0.2 *μ*m^2^/s and 0.06 ± 0.03 (mean ± SD), respectively, and were calculated from the recovery curve by fitting the intensities over time as described in Jönsson et al. (38).

Video S1. Time-lapse of two shrinking cell-SLB contacts due to prolonged imidazole incubation. The scale bar is 8 *μ*m and the time interval between images is 1 minute.

Video S2. Fluorescence recovery after photobleaching (FRAP) measurement on a bilayer functionalised with rCD2-AF647 at a density of 300 molecules/*μ*m^2^. The scale bar is 8 *μ*m and the time interval between images is 2 seconds.

Video S3. Time-lapse of a Jurkat T cell expressing rCD48_T92A_ interacting with an SLB functionalised with 200 molecules/*μ*m^2^ of rCD2-AF647 showing the dynamic nature of the small, round, non-binding regions inside the cell-SLB contact. The scale bar is 8 *μ*m and the time interval between images is 3 minutes.

### Microscopy setup

The proteins were studied in total internal reflection fluorescence mode with a customized inverted Nikon Eclipse Ti microscope (Nikon, Tokyo, Japan) with a motorized *xy*-stage (MLS203; Thorlabs, Newton, NJ), using a 100× oil immersion objective (Plan Apo total internal reflection fluorescence, NA = 1.49; Nikon). A motorized stage was used to change the field of view position, thus imaging various positions of multiple cells contacts on the SLB. The fluorescently labeled proteins were illuminated using an Oxxius LBX diode laser (Lannion, France) operating at 638 nm. The images were collected with a Photometrics Prime 95B sCMOS camera (Tucson, AZ) via *μ*Manager version 1.4 (39).

The FRAP measurements were performed by focusing the incoming light to a delimited area on the sample as well as increasing the laser intensity for 3 s, which resulted in a small region of the sample being bleached. The time of the intensity recovery in this area was subsequently monitored for 45 frames with a time between frames of 2 s.

### Zhu-Golan and image analysis

From the Zhu-Golan analysis, the 2D *K*_d_ for a receptor-ligand binding interaction can be obtained by fitting a line to a plot of *B*/*F* versus *B* × *p*:

(1) $$\frac{B}{F}=\;\frac{N_{\mathrm{tot}\;}\times \;f}{K_{\text{d}}\times S_{\text{cell}}}-\;\frac{B\;\times \;p}{K_{\text{d}}}\;,$$

where *B* and *F* are the density of bound and free proteins, respectively. *N*_t__ot_ × *f* is the total number of mobile receptors on the cell surface, *S*_cell_ is the total surface area of the cell, and *p* is the contact area, *S*_contact_, divided by *S*_cell_ (21). The total surface area was determined from brightfield images using:

(2) $$S_{\text{cell}}=\;4\pi r^{2}\times 1.8\;,$$

where *r* is the radius of the cell, and 1.8 is a factor that takes into account the roughness of the cell surface (40). The area *S*_cell_ was calculated for each cell after the first equilibration step (before imidazole treatment). A similar cell radius, although slightly lower after imidazole treatment, was found for each titration step with average values of 5.3 ± 0.2, 5.1 ± 0.2, 4.9 ± 0.3, and 5.0 ± 0.2 *μ*m (mean ± SD) for the first to the last titration measurement, respectively. Using the radius from the first measurement, this gave *S*_cell_ = 640 ± 170 *μ*m^2^ (mean ± SD). The contact area was obtained by thresholding for high intensity areas and correcting the outline manually for small, round, nonbinding regions inside the cell-SLB contact (Video S3). The 2D *K*_d_ was obtained by the negative reciprocal slope of the fitted line, and the *B* × *p*-axis intercept gave the total density of mobile receptors on the cell.

The mean gray intensity of rCD2 in the contact was measured and converted into total ligand density in the contact (*B* + *F*). The conversion from intensity to ligand density was obtained from single-molecule measurements of the intensity from one rCD2 molecule as described in detail elsewhere (36). The amount of free ligands, *F^∗^,* on the SLB was calculated from the mean gray intensity outside of the cell-SLB contact. However, the free ligand density inside the contact has been found to be lower than the density outside the contact (23,31). This exclusion was measured using a nonbinding molecule in the SLB, 1G4 TCR (see Junghans et al. (27) for details), before and after imidazole treatment. The density of rCD2 in the SLB was 180 and 110 molecules/*μ*m^2^ before and after imidazole treatment, respectively, and the corresponding 1G4 TCR density was 160 and 100 molecules/*μ*m^2^. As previously observed (27), there was no significant dependence on the exclusion with contact size, *p*, below a cell. In addition, the exclusion before and after imidazole treatment was 74 ± 6% and 76 ± 7% (mean ± SD), respectively. This is a nonsignificant difference, and the average exclusion was set to 75% for all ligand titrations. Furthermore, the mobile fraction of the receptors on the cell surface was set to 0.62 as measured previously using FRAP (27). All images were analyzed using Image J (version 1.49V).

## Results

### rCD2 binding rCD48_T92A_ on SLBs with different ligand densities

**Figure 1:**
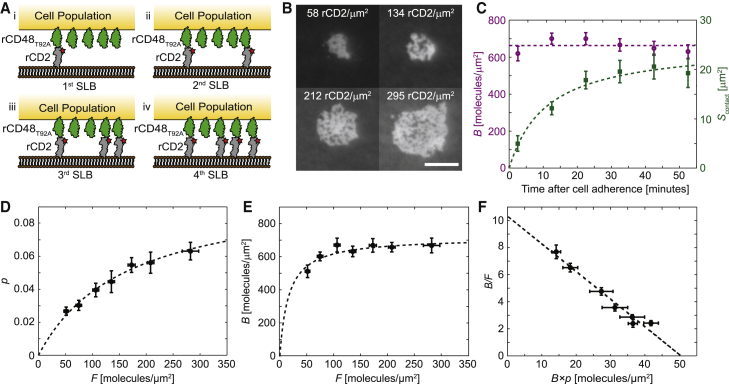
rCD2 binding rCD48_T92A_ on SLBs with different ligand density. (*A*) Schematic illustration showing Jurkat T cells expressing rCD48_T92A_ binding to SLBs with increasing rCD2 density (first to fourth SLB). (*B*) Fluorescence images of cell-SLB contacts at four rCD2 densities in different SLBs. The images were captured 40 min after the cells adhered on the SLB. The scale bar is 8 *μ*m, and the scale is the same for all images. (*C*) The density of bound ligands (*B*; *purple circles*) and cell-SLB contact size (*S*_contact_; *green squares*) as a function of time after a cell adhered on the SLB. The free ligand density in the SLB was 300 molecules/*μ*m^2^. (*D*) Ratio of the contact size to the total cell surface area, *p,* as a function of free ligand density, *F*. (*E*) Steady-state density of bound ligands as a function of free ligand density. (*F*) Zhu-Golan plot of *B*/*F* versus *B* × *p*, which is fitted to Eq. 1 (*dashed line*), resulting in a *K*_d_ of 4.9 ± 0.4 molecules/*μ*m^2^ for the rCD2-rCD48_T92A_ interaction (mean ± SD). Data points represent mean ± SE from 10 (*C*) and 21 (*D*–*F*) different cell-SLB contacts. The dashed lines are fits to either a line or to a Langmuir adsorption isotherm. To see this figure in color, go online.

Jurkat T cells expressing rCD48_T92A_ receptors were added to SLBs functionalized with different densities of fluorescently labeled rCD2 ligands (Fig. 1 *A*). The higher the ligand density, the more rCD2 bound rCD48_T92A_ and thus accumulated in the cell-SLB contact (Fig. 1 *B*). After the cells adhered on the SLB and started forming a cell-SLB contact (Video S4), the density of bound ligands, *B*, almost immediately reached its steady-state value at 600–700 rCD2 molecules/*μ*m^2^, whereas the cell-SLB contact area continued to expand for ∼30 min before leveling out (Fig. 1 *C*). A similar time for the total amount of bound ligands, *B* × *S*_contact_, to reach steady state has previously been observed for wild-type rCD2 cells binding rCD48 in SLBs (22). This means that at this density, the rate of contact area expansion equals the rate with which receptors are diffusing into the contact from the surrounding cell surface to bind free ligands. In addition, the fractional contact area *p* = *S*_contact_/S_cell_ increased steadily in size with free ligand density (Fig. 1 *D*), whereas the average density of bound rCD2 only increased marginally with free ligand density above 50 molecules/*μ*m^2^ (Fig. 1 *E*). The constant bound ligand density would agree with the hypothesis that there is a balance between contact area expansion, whose rate is dependent on the bound density, and the diffusion of receptors into the contact, which depends on the total receptor number. At steady state, the total number of ligand-receptor pairs increases with ligand density, and with an approximately constant bound ligand density, this leads to an increase in contact size as shown in Fig. 1 *D*.

Video S4. Time lapse of four Jurkat T cells expressing rCD48_T92A_ adhering on a rCD2 functionalised SLB and forming cell-SLB contacts. The scale bar is 8 *μ*m and the scale is the same for all images. The time interval between images is 3 minutes.

The relative accumulation of rCD2 in the cell-SLB contacts, *B*/*F*, decreased with higher concentration of free rCD2 in the SLB. This was visualized in a Zhu-Golan plot with *B*/*F* on the *y*-axis and *B* × *p* on the *x*-axis (Fig. 1 *F*). Fitting the data in Fig. 1 *F* to a line according to Eq. 1 gave a 2D *K*_d_ of the rCD2-rCD48_T92A_ interaction of 4.9 ± 0.4 molecules/*μ*m^2^ (mean ± SD). This is similar to previous 2D *K*_d_ values of this interaction measured either at RT or at 37°C (2D *K*_d_ of 6 molecules/*μ*m^2^) (27,32). The fit to Fig. 1 *F* also gave a density of 51 ± 2 mobile rCD48_T92A_ molecules/*μ*m^2^ per cell (mean ± SD), which corresponds to a total number of 53,000 receptors per cell using *S*_cell_ = 640 *μ*m^2^ and assuming *f* = 0.62 (27). This value is comparable in magnitude to an average number of 47,000 rCD48_T92A_ molecules per cell previously obtained for this cell line using flow cytometry (32).

### rCD2 binding rCD48_T92A_ on one SLB and titrating the ligand density with imidazole

**Figure 2:**
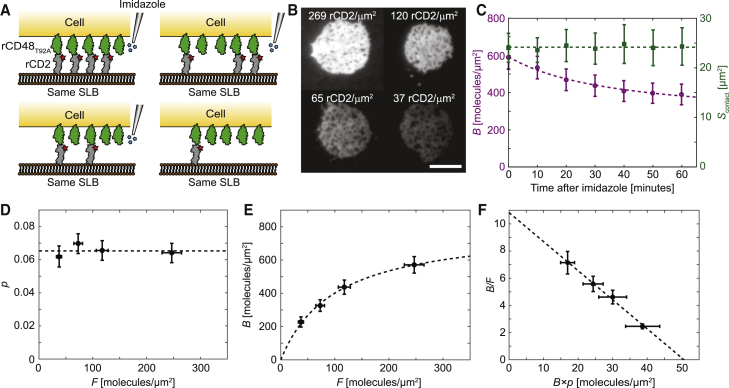
rCD2 binding rCD48_T92A_ by titrating ligand density on the same SLB. (*A*) Schematic illustration showing a Jurkat T cell expressing rCD48_T92A_ binding to an SLB (*top left*) and then subsequently lowering the rCD2 density on the SLB using imidazole. (*B*) Fluorescence images of the same cell-SLB contact at four rCD2 densities in the SLB. The images were captured 40 min after each ligand titration. The scale bar is 8 *μ*m, and the scale is the same for all images. (*C*) The density of bound ligands (*B*; *purple circles*) and cell-SLB contact area (*S*_contact_; *green squares*) as a function of time after the first ligand titration on the SLB. The free ligand density in the SLB was 250 molecules/*μ*m^2^. (*D*) Ratio of the contact size to the total cell surface area, *p,* as a function of free ligand density, *F*. (*E*) Steady-state density of bound ligands as a function of free ligand density. (*F*) Zhu-Golan plot of *B*/*F* versus *B* × *p*, which, fitted to Eq. 1 (*dashed line*), resulted in a *K*_d_ of 4.7 ± 0.2 molecules/*μ*m^2^ for the rCD2-rCD48_T92A_ interaction (mean ± SD). Data points represent mean ± SE from 7 (*C*) and 31 (*D*–*F*) different cell-SLB contacts. The dashed lines are fits to either a line or to a Langmuir adsorption isotherm. To see this figure in color, go online.

In the previous section, multiple SLBs were used with different ligand densities, and averaging over the cell population was performed to obtain information about the rCD2-rCD48_T92A_ interaction. This also means that the accumulation in the contact is not measured on the same cells. An alternative to obtaining different ligand densities in the SLB but still measuring on the same cells is to titrate the ligand density in the SLB, which we do here by adding imidazole (Fig. 2 *A*). Imidazole competes with the polyhistidine-tagged ligands for binding to nickel-chelating lipids on the SLB, and by adjusting the imidazole concentration and the incubation time, the ligand density in the SLB can be accurately adjusted, resulting in different levels of accumulation under the cells (Fig. 2 *B*). It should be noted that the cell contacts will at lower rCD2 densities (*F* ∼10 molecules/*μ*m^2^) start to shrink and eventually detach upon rinsing (Fig. S1; Video S1). All cell-SLB areas contained a small fraction of nonbinding regions corresponding to, on average, 4, 4, 5, and 7% of the total contact area for the first to the last titration step, respectively. Similar nonbinding regions in cell-SLB contacts have been observed in other studies and systems (26,41,42), both at RT (32) and at 37°C (27), and has been suggested to be influenced by membrane fluctuations (17). It could also be that some of the normally undulating cell surface becomes “trapped” upon binding to the flat SLB.

The time to reach a steady state after imidazole had been added and rinsed off was similar to the case with multiple bilayers, 30–40 min; however, in this case, it was the density of bound ligands, *B*, that changed with time, whereas *S*_contact_ remained constant on average (Fig. 2 *C*; Fig. S2). Similarly, the contact/cell area ratio changed only slightly with a varying free ligand density after imidazole treatment (Fig. 2 *D*; Fig. S3), whereas *B* decreased as the ligand density was lowered following the shape of a Langmuir adsorption isotherm (Fig. 2 *E*). When fitting the data in Fig. 2 *E* to Eq. 1, the slope of the fit gave a 2D *K*_d_ of 4.7 ± 0.2 molecules/*μ*m^2^ (mean ± SD) and a mobile density, *N*_tot_×*f*/*S*_cell_, of 51 ± 1 rCD48_T92A_ molecules/*μ*m^2^ corresponding to a total number of 53,000 receptors per cell; both values are within the statistical uncertainty of the values obtained using multiple SLBs. Thus, changing the ligand density using imidazole does not affect the binding affinity of the interaction under these conditions. It is worth emphasizing the similarity of the Zhu-Golan plots in Fig. 1 *F* and Fig. 2 *F*, despite having significantly different *B* versus *F* curves (Figs. 1 *E* and 2 *E*). Thus, the 2D *K*_d_ appears independent of the density of bound receptors in the range shown in Fig. 2 *E*, indicating that membrane fluctuations do not influence the 2D *K*_d_ significantly for this system and density range (16).

### Single-cell binding of rCD2 to rCD48_T92A_

**Figure 3:**
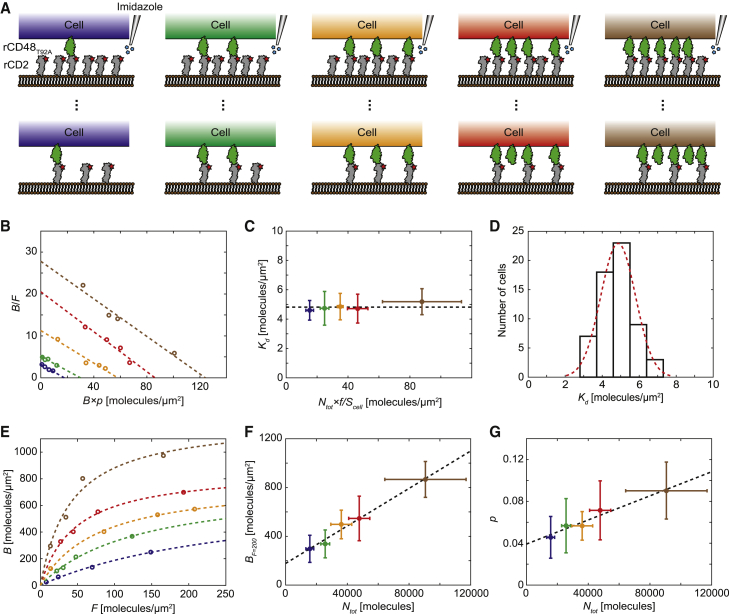
Single-cell measurements of rCD2 binding rCD48_T92A._ (*A*) Binding of individual cells, with a varying amount of receptors, to a single SLB in which the amount of ligands in the SLB is titrated using imidazole. (*B*) Zhu-Golan plots for five representative cells with different receptor densities. The dashed lines are linear fits to the data (Eq. 1). (*C*) The dependence of 2D *K*_d_ on receptor density. Each data set corresponds to 12 cells, grouped with respect to the total number of rCD48_T92A_ receptors per cell obtained via the individual Zhu-Golan plots. The dashed line corresponds to the average *K*_d_ value. (*D*) Histogram of single-cell 2D *K*_d_s from 60 individual cells (from seven separate SLBs). The dashed line is a Gaussian fit to the data with a mean value of 4.9 molecules/*μ*m^2^. (*E*) Density of bound rCD2 molecules as a function of free rCD2 density for the five representative cells in (*B*). The dashed lines are fits of the data to a Langmuir adsorption isotherm. (*F*) Density of bound rCD2 at *F* = 200 molecules/*μ*m^2^ for cells with different numbers of rCD48_T92A_ receptors per cell. Each data set corresponds to 12 cells, grouped with respect to the total number of rCD48_T92A_ receptors per cell obtained via the individual Zhu-Golan plots. The dashed line is a linear fit to the data. (*G*) The dependence of the relative contact area (*p* = *S*_contact_/*S*_cell_) on the number of rCD48_T92A_ receptors per cell. Each data set corresponds to 12 cells, grouped with respect to the total number of rCD48_T92A_ receptors per cell obtained via the individual Zhu-Golan plots. The dashed line is a linear fit to the data. Data points represent mean ± SE in all figures. To see this figure in color, go online.

It was shown in the previous section that the average 2D *K*_d_ over the cell population could be obtained by titrating the amount of rCD2 in a single SLB using imidazole. Whereas traditionally such data had been obtained by using separate SLBs with different ligand concentrations, this approach has the advantage that the binding behavior of individual cells can be monitored (Fig. 3 *A*). In particular, this allows for a Zhu-Golan plot to be made for each individual cell and a corresponding 2D *K*_d_ and receptor density for each cell to be obtained (Fig. 3 *B*). In Fig. 3 *C*, five representative cell groups with different mobile receptor densities, *N*_tot_×*f*/*S*_cell_, are shown, from an average of 18 rCD48_T92A_ per *μ*m^2^ (*blue*) to an average of 124 rCD48_T92A_ per *μ*m^2^ (*brown*), as determined from their *x*-intersect in the Zhu-Golan plot. Although the receptor density varies by almost an order of magnitude and thus also the accumulation, there was no significant difference in the obtained 2D *K*_d_ as shown in Fig. 3 *C*, where, in total, 60 cells have been grouped into five separate groups based on *N*_tot_. The average 2D *K*_d_ in the cell population is also rather narrow with an average value and SD of 4.9 ± 0.9 molecules/*μ*m^2^, approximately following a Gaussian histogram (Fig. 3 *D*). The order of magnitude spread observed in the accumulation and density of bound ligands here, and in other studies (26), is thus more likely because of differences in the number of available receptors on the cells and not because of a difference in cell affinities. Because cells with more receptors give a stronger signal, this could also allow for a more accurate affinity measurement of weakly binding receptor-ligand pairs by restricting the analysis to cells with the highest number of bound ligands.

Fig. 3*E* shows how the density of bound ligands varies with the ligand density for the five representative cells in Fig. 3 *B*. The *B* versus *F* curves can all be described by a Langmuir adsorption isotherm but with varying slopes and steady-state values. The latter can be visualized by plotting *B* at *F* = 200 molecules/*μ*m^2^ (*B*_F = 200_), approximately corresponding to the steady-state accumulation before the first titration (cf. Fig. 1 *E*), as a function of the number of rCD48_T92A_ receptors per cell determined from the Zhu-Golan plots (Fig. 3 *F*). The data have been sorted and averaged into five separate groups, with respect to *N*_tot_, and it can be observed that *B*_F = 200_ increases linearly with *N*_tot_. This agrees with the hypothesis presented previously when discussing the bound density on separate bilayers that the steady-state value of *B* depends on the rate of receptors diffusing into the contact from outside. As the amount of receptors on the cell increases, the rate of receptors diffusing into the contact will also increase, leading to a higher steady-state value of *B*. Finally, also the contact size increases approximately linearly with the receptor number in the studied interval (Fig. 3 *G*). This is in agreement with what has been previously observed in which the cell contact increased with the total number of bound ligands in the contact (27,42). Because more receptors lead to more bound ligands, this motivates the increase in contact size for cells with a higher receptor number.

## Discussion

We present a single-cell method to measure 2D binding affinities in cell contacts. This method extends the classical Zhu-Golan method by titrating the amount of free ligands in a cell-SLB contact using imidazole. The effect of imidazole on the binding affinity of the cell population was first investigated and the similarities of the Zhu-Golan plots obtained by using imidazole and by the traditional Zhu-Golan approach indicate that imidazole did not influence the binding significantly. Thus, from the resulting ligand-receptor accumulation, we could determine the affinity of the interaction and the number of cell receptors. The single-cell method has a number of advantages and implications as discussed in turn below.

Multiple cell measurements are needed for the traditional Zhu-Golan analysis to obtain accurate average values because of the inherent spread in accumulation under the different cells. Because a different batch of cells and a different SLB are needed for each new data point, there is also the possibility that the cells and the system might have changed between the measurements, which adds an extra uncertainty to the analysis. In contrast, in the single-cell analysis, it is in principle sufficient to measure a single-cell contact at two different concentrations to get a similar, or better, accuracy in the binding affinity as with the traditional Zhu-Golan analysis, which often requires the analysis of hundreds of cells. This can reduce analysis time significantly because accurately finding the contour of each contact and measuring the accumulation and contact size is often a time-consuming task that requires manual validation, especially for weaker binding interactions. It is worth noticing that titrating with imidazole, thus going from high to low ligand density, appears to be important. Going the opposite direction, from low to high, by adding more ligands was previously performed by us (27). However, whereas it was found that this approach gave a similar population-averaged value of *K*_d_ compared with the traditional Zhu-Golan method, it was not possible for us to obtain single-cell *K*_d_ values because the spread among the Zhu-Golan curves was very large, with some cells even giving negative *K*_d_ values (data not shown). The reason why going from high to low density is more robust than going from low to high can only be speculated about, but could, potentially, be due to the contact growth, thus not reaching equilibrium until at much later times in the latter case.

Compared with the traditional Zhu-Golan method, the single-cell measurements require the cells to be bound for a longer time to the SLB in order for the accumulation to reach equilibrium after each titration step. Whereas this prolonged attachment did not appear to negatively affect the here-used Jurkat T cells, other cell types might behave differently. Another difference between the single-cell method compared with the traditional Zhu-Golan method is that the cell-SLB size remains approximately constant when titrating the ligand concentration from high to low above a certain density of the order of 10 molecules/*μ*m^2^ (Fig. 2 *D*). In contrast, for cells on different SLBs, the size of the cell-SLB contact increased with ligand density (Fig. 1 *D*). This shows that there is a hysteresis in *S*_contact_ versus *F*. Thus, it takes a higher ligand density to expand the cell-SLB contact than it takes to keep the contact area stationary in size. This makes it possible to have larger contacts even for low ligand concentrations when titrating with imidazole. It was also found that after first contact with the SLB, the cell-SLB contacts expanded at an approximately constant density of bound ligands, but that this density varied from cell to cell (Fig. 1 *C*).

From the single-cell analysis, it was found that the density of bound rCD2 at *F* = 200 molecules/*μ*m^2^, corresponding to the condition before imidazole addition, scaled linearly with receptor number but had a lower threshold of around 200 bound molecules/*μ*m^2^ (Fig. 3 *F*). This could possibly correspond to the lowest density of bound receptors that can promote cell-SLB contact growth for this system. As a comparison, with an average density of mobile rCD48_T92A_ receptors of 50 molecules/*μ*m^2^ and with a 2D *K*_d_ of 5 molecules/*μ*m^2^, this means that to reach 200 bound molecules/*μ*m^2^ and start expanding, the free ligand density, *F*, needs to be at least 20 molecules/*μ*m^2^. This density is similar to the minimal density of ligands required for cell-SLB contacts to form in this and other studies on similar systems (21,27).

As demonstrated in this and previous studies, there is commonly a significant spread in the cell-SLB accumulation among different cell-SLB contacts (26,27). This can be influenced by the inherent spread in receptor density over the cell population but also by the heterogeneity in the binding capacity among the cell population (43). We here find that the spread in rCD2-rCD48_T92A_ 2D affinities among the cell population is relatively small and that the large differences in accumulation stem from varying receptor densities on the cells. A rather narrow spread in 2D *K*_d_ has also previously been observed for other binding pairs using micropipette-based adhesion assays (4,44), whereas single-molecule fluorescence studies on the TCR-MHC interactions have shown a larger spread among the cells (28,29); however, it cannot be ruled out that at least part of this spread is due to the single-cell accuracy of the methods used in the latter studies. Whereas a low spread in 2D affinity within the cell population might be true when there is only one type of ligand-receptor pair present, there are multiple different protein-protein interactions across cell contacts in vivo (45, 46, 47, 48). It has been shown that mixtures of different ligand-receptor pairs can either stabilize and align the cell-cell interface for binding or impair binding, depending on the properties of the different proteins (27,49). An important factor here is the relative density of the different proteins, a factor that could vary among the cell population, which in turn could give rise to a difference in binding affinity. It would be valuable to only reduce the concentration of one of the protein ligands in the SLB to be able to study this in more detail, which can be done by using a different anchor to the SLB for these proteins than a polyhistidine tag, for example using a biotin or SNAP tag (50,51). The spread in accumulation in the cell population can also potentially be exploited for situations in which the binding is weak. Here, analyzing cells with the highest receptor density can give an almost order of magnitude stronger signal compared with the average binding signal. Having single-cell information will, in addition, overcome the problem of not all cells binding and forming contacts with the SLB at low ligand densities (22). This can give rise to an apparent shift in the Zhu-Golan plot and thereby influence the effective affinity. The single-cell Zhu-Golan method does not suffer from this disadvantage because it analyzes the same cell contact.

## Conclusions

In summary, measuring the 2D affinity of single cells can considerably reduce both experiment and analysis time because, instead of having to analyze hundreds of cells to get an accurate average accumulation, a similar accuracy in affinity can be obtained from studying the contact formed between a single cell and an SLB and from titrating the ligand concentration in this contact using imidazole. It was also found that, in this case, the spread in 2D affinity was small within the cell population and did not depend on the number of receptors expressed by the different cells. This, together with the possibility of selecting cells within the population expressing a high receptor density and thus producing the most accumulation, opens up for a rapid and accurate determination of weakly binding ligand-receptor affinities, avoiding the inherent spread in receptor densities influencing classical methods. It would also be feasible to use combinations of ligands with different anchors that would make it possible to reduce only one type of ligand while keeping the concentration of the others fixed, which would allow for more complex, but realistic, affinity measurements to be performed.

## Author contributions

Designed research, M.C. and P.J.; performed research, M.C.; analyzed data, M.C. and T.D.; contributed with resources, V.J., A.M.S., and S.J.D.; and wrote the manuscript, all authors.

## Supporting material

Document S1. Figs. S1–S3

Document S2. Article plus supporting material
