# Supplementary material for: Single-cell measurements of two-dimensional binding affinity across cell contacts
Source: Biophys J. 2021 Oct 13;120(22):5032–40. doi: 10.1016/j.bpj.2021.10.010 (PMC8633712; doi:10.1016/j.bpj.2021.10.010)
Supplement: Document S1. Figs. S1–S3 [file mmc1.pdf]

Biophysical Journal, Volume *120*

Supplemental information

Single-cell measurements of two-dimensional binding affinity across cell contacts

Manto Chouliara, Victoria Junghans, Tommy Dam, Ana Mafalda Santos, Simon J. Davis, and Peter Jönsson

Supporting Figures

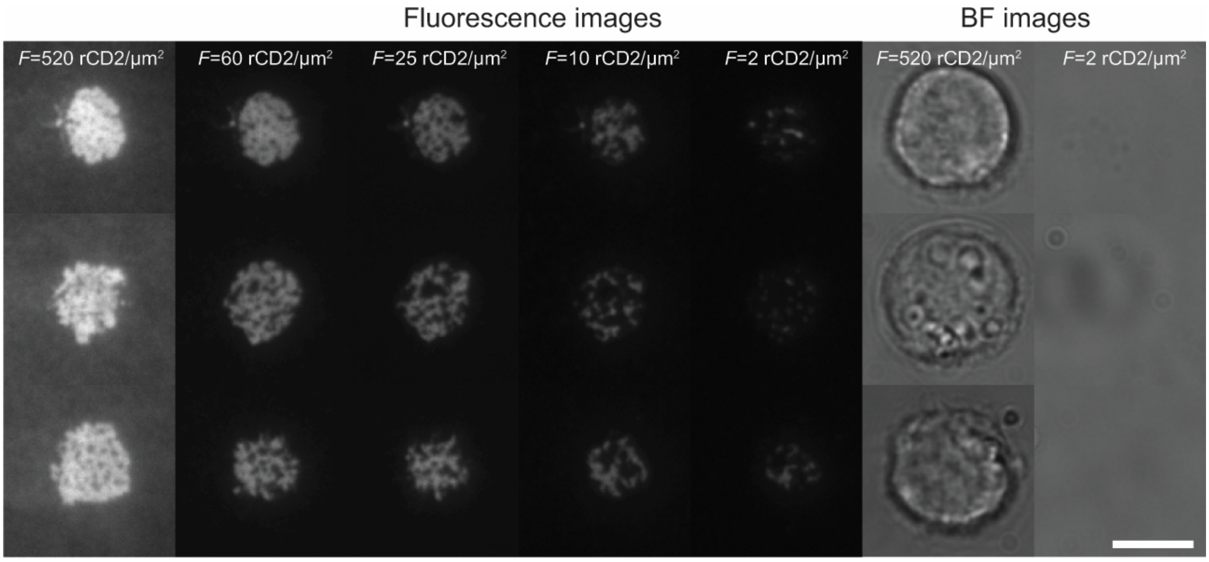

Figure S1. Extensive ligand titration using imidazole leads to shrinking cell-SLB contacts and cell detachment. Fluorescence and brightfield (BF) images of three cell-SLB contacts at five rCD2 densities in the SLB. Initial (left) and subsequent (right) snapshots of cell-SLB contacts due to incubation with imidazole. Rinsing with imidazole-free buffer resulted in the detachment of the cells from the SLB (see BF images to the right). The scale bar is 8 μm and the scale is the same for all images.

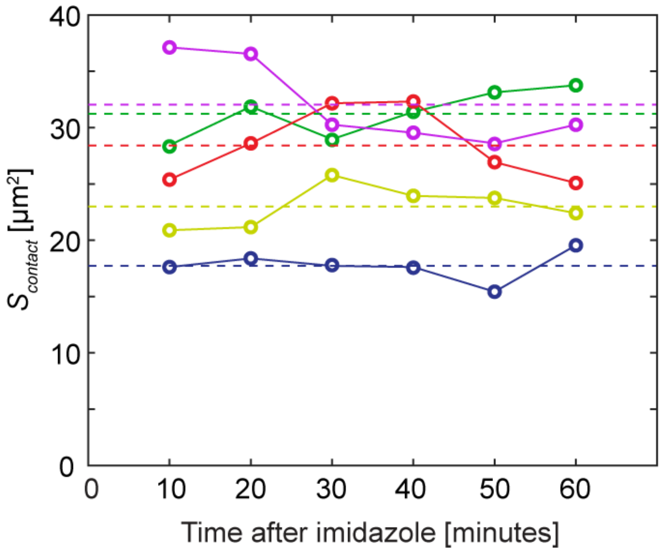

Figure S2. The cell-SLB contact area, S_{contact}, as a function of time after ligand titration for five representative cells. The data corresponds to cells after the first imidazole step.

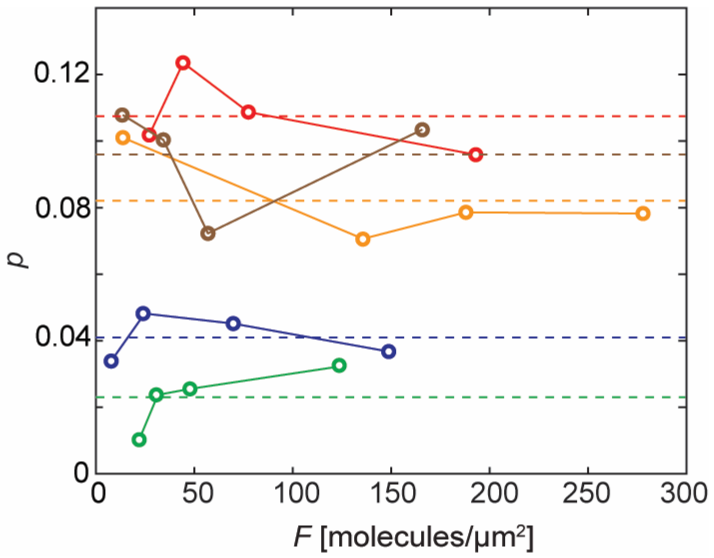

Figure S3. The ratio of the contact size to the total cell surface area, *p*, as a function of free ligand density, *F*, for the five representative cells presented in Figures 3B and 3E.
